# Novel mutations in *ATP7B* in Chinese patients with Wilson's disease and identification of kidney disorder of thinning of the glomerular basement membrane

**DOI:** 10.3389/fneur.2023.1231605

**Published:** 2023-08-23

**Authors:** Hongliang Xu, Hanyu Lv, Xin Chen, Yajun Lian, Guolan Xing, Yingzi Wang, Ruimin Hu

**Affiliations:** ^1^Department of Neurology, The First Affiliated Hospital of Zhengzhou University, Zhengzhou, Henan, China; ^2^Department of Nephrology, The First Affiliated Hospital of Zhengzhou University, Zhengzhou, Henan, China; ^3^Department of Renal Electron Microscopy, The First Affiliated Hospital of Zhengzhou University, Zhengzhou, Henan, China

**Keywords:** Wilson's disease, *ATP7B* gene, novel mutations, renal pathological, liver transplantation

## Abstract

**Introduction:**

Wilson's disease is an autosomal recessive disorder caused by *ATP7B* pathogenic mutations. The hallmark of this disorder mainly consists of liver involvement, neurologic dysfunction and psychiatric features. In addition, the kidneys can also be affected by excessive copper deposition.

**Methods:**

A total of 34 patients clinically diagnosed with WD were recruited. They underwent *ATP7B* gene sequencing and clinical data of symptoms, examination, and treatment were collected. Moreover, renal pathology information was also investigated.

**Results:**

We identified 25 potentially pathogenic *ATP7B* variants (16 missense, 5 frameshift, 3 splicing variants and 1 large deletion mutation) in these 34 WD patients, 5 of which were novel. In our cases, the most frequent variant was c.2333G>T (R778L, 39.06%, exon 8), followed by c.2621C>T (A874V, 10.94%, exon 11) and c.3316G>A (V1106I, 7.81%, exon 11). Furthermore, we described the thinning of the glomerular basement membrane as a rare pathologically damaging feature of Wilson's disease for the first time. Additionally, two patients who received liver transplant were observed with good prognosis in present study.

**Discussion:**

Our work expanded the spectrum of *ATP7B* variants and presented rare renal pathological feature in WD patients, which may facilitate the development of early diagnosis, counseling, treatment regimens of WD.

## 1. Introduction

Hepatolenticular degeneration was first described by Wilson in 1912 and is also called Wilson's disease (WD). It is a rare autosomal recessive disease, which is characterized by hepatic, neurological, and psychiatric symptoms (1). The hallmark of this disorder is excessive copper (Cu) deposition in the liver and the brain, manifested as cirrhosis, neurologic dysfunction, and psychiatric features (2). Renal damage, olfactory dysfunction, and arrhythmias may also be present (3). Diagnosis mainly depends on clinical features and laboratory parameters, including serum ceruloplasmin, 24-h urinary copper excretion, and total serum copper. As a matter of fact, mutational analysis is also necessary for diagnosis (4). Even though the symptoms are complex and not fully understood, there are some major pathways for the general management of WD: anti-copper agents (chela-tors or zinc salts), liver transplantation, and supportive treatment (5, 6).

In 1993, mutations in the *ATP7B* gene were identified as the cause of WD, which is located in chromosome 13. *ATP7B* encodes an eight-transmembrane-domain protein consisting of 1465 amino acids, which is associated with copper transport (4, 7). As a multi-domain protein, *ATP7B* plays a critical role in transferring Cu across the cell membrane using energy generated by ATP hydrolysis. The functions of *ATP7B* include transporting copper into the trans-Golgi network (TGN) for incorporation into ceruloplasmin and trafficking from TGN to endocytic vesicles in response to the elevation of intracellular copper concentration (8, 9). To date, more than 800 *ATP7B* mutations have been identified as disease-causing mutations (4, 7). Recent advances in the identification of mutations in the *ATP7B* gene have greatly improved the accuracy of WD diagnosis and treatment in affected patients and their siblings.

The genetic defect in *ATP7B* usually results in liver injury and neuropsychiatric involvement, while kidney disease is a presenting feature in only approximately 1% of patients (10). Clinically, excess copper in renal parenchyma may cause renal dysfunction, which manifests as hematuria, proteinuria, and so on (4, 11). However, to date, little has been described about the pathological features of kidney damage in WD patients. In the present study, we conducted genetic identification and clinical analysis of 34 WD patients in a Chinese cohort. This study aimed to broaden the previously established *ATP7B* mutation spectrum; investigate pathological characteristics of patients with kidney involvement; and analyze the symptoms, examination, and treatment of WD patients.

## 2. Materials and methods

### 2.1. Patients and data collection

This retrospective study was designed in accordance with the principles of the Declaration of Helsinki and was approved by the Ethics Committee of the First Affiliated Hospital of Zhengzhou University. Informed consent was obtained from all recruited subjects or their parents.

A total of 34 WD patients (22 male and 12 female patients; onset age was from 3 to 50 years) from China were recruited between January 2019 and October 2022 at the First Affiliated Hospital of Zhengzhou University in this study. The clinical data for each participant were obtained from the medical records.

The diagnosis of WD was based on a combination of the medical history, physical examination results, laboratory test data, and imaging results. Laboratory tests include serum ceruloplasmin, biochemical liver tests, and renal function tests. Imaging results include liver ultrasound and brain magnetic resonance imaging (MRI).

In addition, renal puncture biopsy reports also form part of the clinical data. Renal pathology data include light microscopic structures, electron microscopic structures, and immunofluorescence results.

### 2.2. Mutation screening and genetic analysis

An average of 4 ml of peripheral blood sample was taken from each participating individual for mutation analysis. All 21 exons were amplified by polymerase chain reaction (PCR) and were then sequenced using high-throughput sequencing (“next-generation” sequencing technology, NGS). PCR products were analyzed using the automated DNA sequencer to detect the disease-causing mutations and single nucleotide polymorphisms.

To further test whether large rearrangement occurred, we performed multiplex ligation-dependent probe amplification (MLPA) in a clinically diagnosed patient who was detected with only one pathogenic variant using NGS.

All detected sequences were compared to the reference (NM_000053.3) from NCBI. The Genome Analysis Toolkit 4 (GATK4) pipeline was used to perform variant calling. The putative effects of each variant were predicted using bioinformatics software, namely Polyphen-2 and Mutation Taster. When identifying the novel sequence variant, gene sequencing results were compared with published known variants deposited in ClinVar (12). The pathogenicity of the variant was assessed according to the American College of Medical Genetics and Genomics (ACMG) guidelines.

## 3. Results

### 3.1. Clinical features

The main clinical data of the affected subjects are summarized in Table 1. Demographics include 12 (35.3%) female patients and 22 (64.7%) male patients with ages ranging from 5 to 55 years old. Age at onset of first clinical symptoms ranged from 3 to 50 years, and the mean age was 21 years old.

**Table 1:** Wilson's disease patient clinical data summary.

**Patient**	**Sex**	**Age of onset**	**Phenotype**	**Serum ceruloplasmin**	**ALT**	**AST**	**Mutation**	**Het/Hom**
Normal				15–45 mg/dl	< 40 U/L	< 40 U/L		
Patient 1	Male	12	Hepatic, Neurologic	3.2 mg/dl	70 U/L	105 U/L	c.2621C>T; 1 large deletion mutation	Het
Patient 2	Male	18	Renal	2.1 mg/dl	12 U/L	14 U/L	c.2333G>T; c.3659-3660insTGA	Het
Patient 3	Female	10	Hepatic	2.7 mg/dl	41 U/L	74 U/L	c.2333G>T; c.2333G>T	Hom
Patient 4	Male	50	Hepatic, Neurologic	10.2 mg/dl	53 U/L	53 U/L	c.3316G>A; c.3517G>A	Het
Patient 5	Male	8	Hepatic	0.2 mg/dl	46 U/L	51 U/L	c.3884C>T	n.a
Patient 6	Female	5	Hepatic	2.8 mg/dl	146 U/L	77 U/L	c.2333G>T; c.2621C>T	Het
Patient 7	Female	22	Neurologic	0.9 mg/dl	16 U/L	23 U/L	c.2333G>T; c.2590_2593dup	Het
Patient 8	Female	20	Neurologic	7.6 mg/dl	9 U/L	21 U/L	c.2333G>T; c.2333G>T	Hom
Patient 9	Male	17	Neurologic	2.1 mg/dl	10 U/L	14 U/L	c.2333G>T; c.3646G>A	Het
Patient 10	Male	9	Renal	2.0 mg/dl	196 U/L	77 U/L	c.2621C>T; c.3724G>A	Het
Patient 11	Male	30	Hepatic, Neurologic	4.1 mg/dl	20 U/L	33 U/L	c.2333G>T; c.3316G>A	Het
Patient 12	Female	14	Hepatic	2.1 mg/dl	18 U/L	27 U/L	c.2333G>T; c.2333G>T	Hom
Patient 13	Male	37	Neurologic	6.8 mg/dl	19 U/L	24 U/L	c.3316G>A; c.3700del	Het
Patient 14	Male	37	Hepatic	13.8 mg/dl	39 U/L	55 U/L	c.2333G>T; c.2494A>C	Het
Patient 15	Male	14	Neurologic	0.7 mg/dl	17 U/L	23 U/L	c.2333G>T; c.2975C>T	Het
Patient 16	Male	14	Neurologic	2.8 mg/dl	30 U/L	53 U/L	c.2333G>T; c.2333G>T	Hom
Patient 17	Male	30	Hepatic	6.0 mg/dl	42 U/L	44 U/L	c.2383C>T; c.3700del	Het
Patient 18	Male	5	Neurologic	4.6 mg/dl	12 U/L	28 U/L	c.2333G>T	n.a
Patient 19	Female	46	Hepatic	4.9 mg/dl	13 U/L	23 U/L	c.2333G>T; c.3593T>C	Het
Patient 20	Male	33	Neurologic	2.2 mg/dl	11 U/L	23 U/L	c.2621C>T; c.4176dup	Het
Patient 21	Male	12	Hepatic	3.9 mg/dl	55 U/L	91 U/L	c.2333G>T; c.2267C>G	Het
Patient 22	Male	3	Asymptomatic	2.2 mg/dl	217 U/L	143 U/L	c.2333G>T; c.2333G>T	Hom
Patient 23	Male	32	Hepatic	6.0 mg/dl	30 U/L	41 U/L	c.3316G>A; c.3517G>A	Het
Patient 24	Male	20	Neurologic	3.7 mg/dl	30 U/L	15 U/L	c.2975C>T	n.a
Patient 25	Male	19	Hepatic, Neurologic	8.1 mg/dl	20 U/L	24 U/L	c.1707+5G>A; c.2333G>T	Het
Patient 26	Female	26	Hepatic	4.5 mg/dl	71 U/L	132 U/L	c.2333G>T; c.3903+2T>G	Het
Patient 27	Female	17	Neurologic	4.0 mg/dl	25 U/L	41 U/L	c.1708-1G>C; c.2333G>T; c.1168A>G	Het
Patient 28	Female	32	Neurologic	3.5 mg/dl	24 U/L	17 U/L	c.2621C>T; c.2975C>T	Het
Patient 29	Male	33	Hepatic, Neurologic	11.7 mg/dl	29 U/L	44 U/L	c.2621C>T; c.2755C>G	Het
Patient 30	Female	19	Neurologic	3.2 mg/dl	14 U/L	20 U/L	c.3316G>A; c.2790_2792del	Het
Patient 31	Male	17	Neurologic	6.0 mg/dl	13 U/L	22 U/L	c.2333G>T; c.2621C>T	Het
Patient 32	Male	29	Neurologic	13.0 mg/dl	38 U/L	28 U/L	c.3451C>T	n.a
Patient 33	Female	11	Neurologic	3.4 mg/dl	11 U/L	24 U/L	c.2662A>C; c.2662A>C	Hom
Patient 34	Female	16	Neurologic	3.0 mg/dl	6 U/L	15 U/L	c.2333G>T; c.2975C>T	Het

n.a, not applicable; Het, heterozygous; Hom, homozygous.

Among the 34 WD patients, the most frequent initial symptom was limb tremor. Another common initial symptom in our population was anorexia. Clinical manifestations of kidney injury apparent in the early stages of the disease were a feature in two individuals. Over the course of the disease, dysphonia, totter, abdominal distension, and edema occurred at a later stage. In 10 out of 34 individuals, hepatic manifestations were present, and neuropsychiatric symptoms were seen in 16 out of 34 patients, with mixed presentation (both hepatic and neuropsychiatric features) documented in five out of 34 patients. Of the 34 patients, one was categorized as an asymptomatic subtype, whose clinical abnormalities were elevated transaminases. In addition, two cases (patients 2 and 10) demonstrated predominantly renal involvement. The clinical features of patient 2 mainly included proteinuria and elevation of blood pressure. Patient 10 was diagnosed with hepatic dysfunction during a medical examination at the age of nine, whose predominant symptom (nocturnal polyuria) appeared until 11 years of life.

### 3.2. Renal pathology

**Figure 1:**
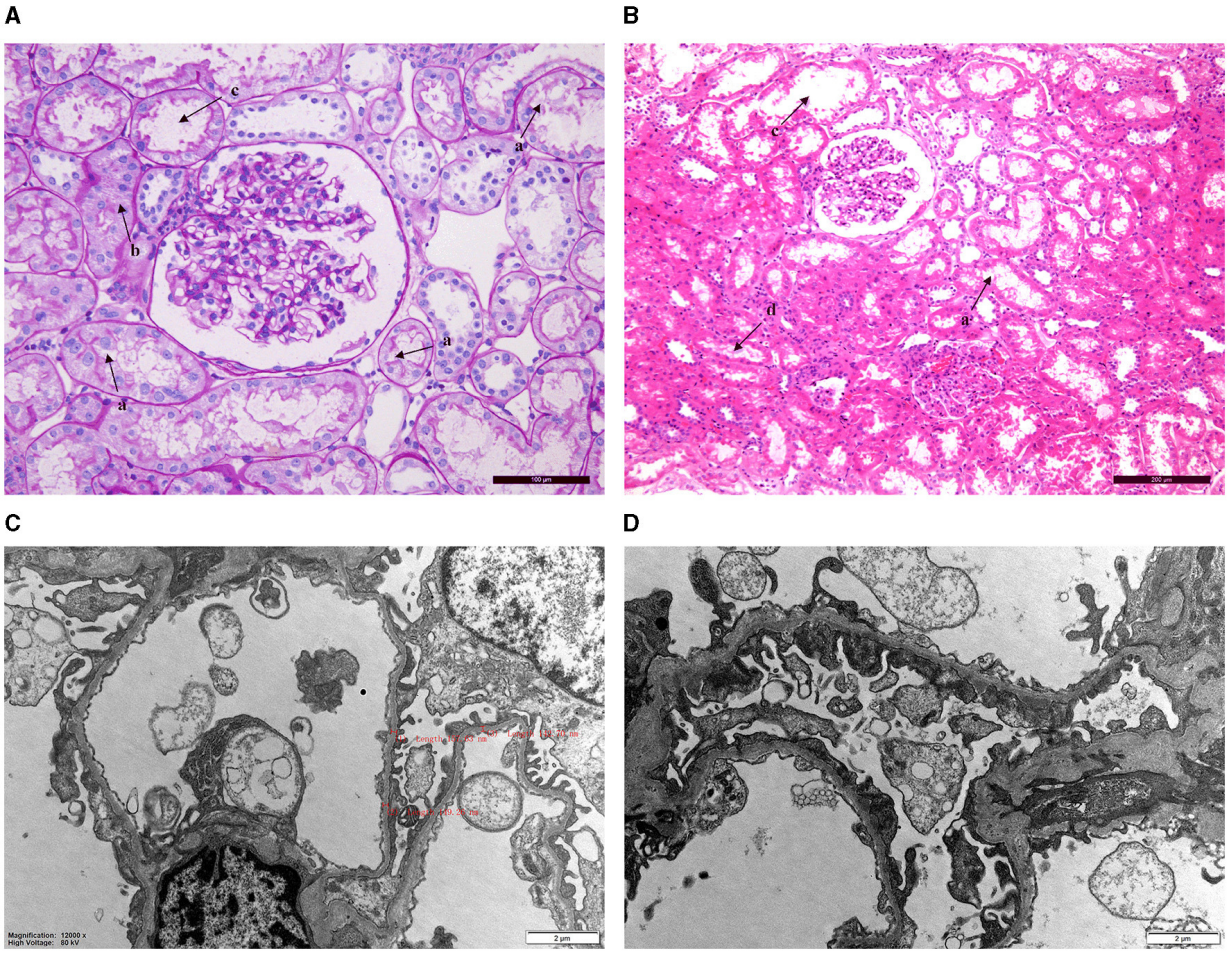
Renal pathology biopsy findings for patient 2. **(A, B)** Light microscopy. a: Vacuolation degeneration of renal tubular epithelial cells. b: Granular degeneration and unclear cell boundaries. c: Small focal tubular dilatation. d: Deposition of granular material. **(C, D)** Electron microscopy revealed thinning of the basement membrane (thickness: 136–204 nm) and foot process fusion of glomerular visceral epithelial cells.

Patient 2 underwent a pathological biopsy of the kidney (Figure 1). Under light microscopy, it revealed vacuolation and granular degeneration of renal tubular epithelial cells, and small focal tubular dilatation; deposition of granular material in segmental capillary walls was also noted (Figures 1A, B). Electron microscopy showed foot process fusion of glomerular visceral epithelial cells and scattered deposits of electron-dense material under the segmental epithelium. Notably, under electron microscopy, we observed diffuse thinning of the basement membrane (Figures 1C, D).

### 3.3. Laboratory results, liver ultrasound, and brain imaging

When the laboratory data of the patients were evaluated (Table 1), it was found that elevated liver enzymes occurred in 14 out of 34 patients. Decreased serum ceruloplasmin was present in all of the 34 patients, with the serum ceruloplasmin level ranging from 0.17 to 13.84 mg/dl. The Kayser–Fleischer ring (K–F ring) was seen in 13 patients (13 out of 20, 65.0%), which was caused by copper accumulation in the Descemet membrane (the basement membrane of the corneal epithelium). The age range of patients who had the K–F ring was from 8 to 43 years old.

In total, 21 out of 30 patients who had a brain MRI (of a total of 34 patients) demonstrated abnormalities, and the basal ganglia is the most commonly affected site. In the current cohort, 17 cases (80.9%) showed a symmetric abnormal signal in the basal ganglia (globus pallidus, red nucleus, substantia nigra, and putamen). The pons was reported in seven patients, thalamus in five cases, and mesencephalon in four cases. In addition, in this study, some atypical MRI findings showed that abnormalities existed in the cerebellum and corpus callosum. Imaging abnormality of liver ultrasound (US) was observed in all cases except in patient 18. Even though the initial abdominal ultrasound results of two cases were normal, follow-up examinations of patients 16 and 30 suggested hepatic diffused lesions. The mean time from the onset of symptoms to the emergence of diffuse echogenic changes in the liver was 23.5 months in these two subjects.

### 3.4. Treatment

Most of the patients (17 out of 34) were treated with zinc salts, and approximately half of the patients (14 out of 34) were treated with penicillamine. In addition, in the present cohort, of those patients with liver involvement, two pediatric patients were treated with liver transplantation. The mean follow-up period was 20 months. The vascular patency suggested by ultrasonography of the transplanted liver proved the success of the operation. Two children suffered from abdominal distention and edema of the lower limbs before the operation, but these symptoms had been improved considerably in our follow-up study. Furthermore, the biochemical indicators returned to normal range after liver transplantation, indicating that the disease was in remission. The serum ceruloplasmin levels of two patients were 0.17 mg/dl and 4 mg/L before surgery and 23.8 6 and 27.38 mg/L at the time of reexamination 1 month after surgery. The serum ceruloplasmin levels of two patients were 0.17 mg/dl and 4 mg/L before surgery and 23.86 and 27.38 mg/L at the time of reexamination 1 month after surgery. After 1 month of liver transplantation, transaminases of both patient 5 (Alanine aminotransferase: from 46 to 30 U/L; Aspartate aminotransferase: from 51 to 26 U/L) and patient 21 (Alanine aminotransferase: from 89 to 15 U/L; Aspartate aminotransferase: from 123 to 15 U/L) were back to normal. Meanwhile, the albumin levels also increased on average 1 month postoperatively (from 28.2 to 48.3 mmol/L in patient 5 and from 21.8 to 44.5 mmol/L in patient 21).

### 3.5. Genetic analysis

**Figure 2:**
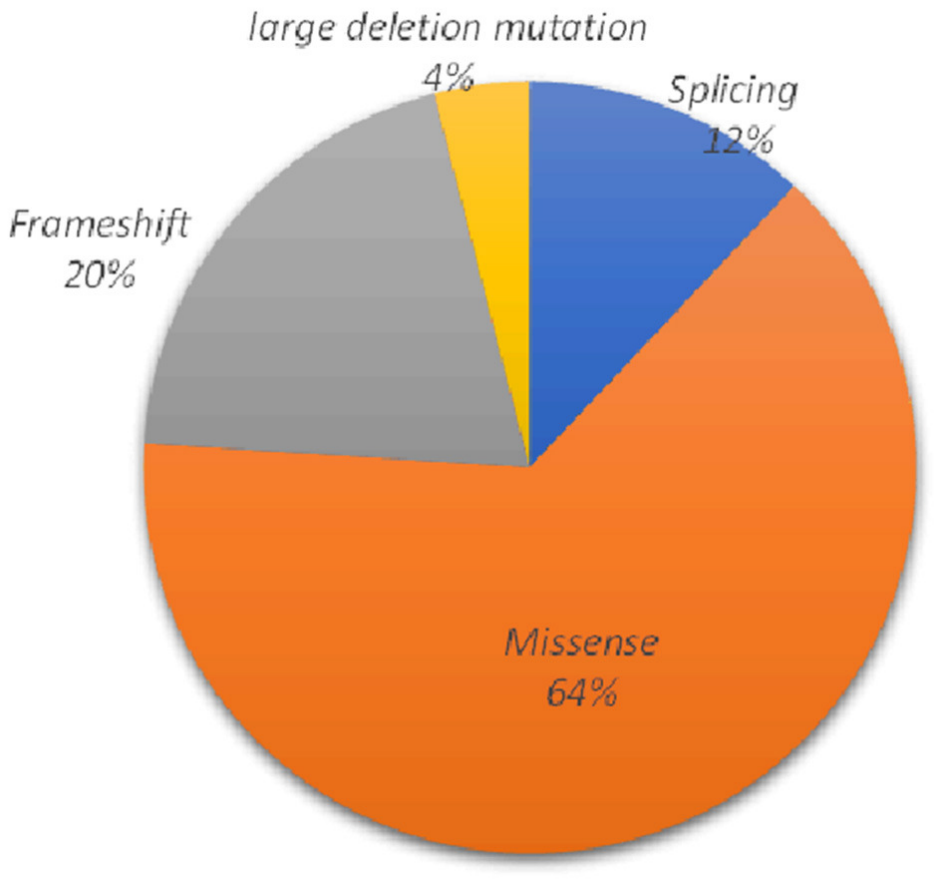
Various mutants identified in this study and their proportions.

As summarized in Table 2, 25 underlying pathogenic mutations, excluding 1 synonymous variant (c.2310C >G) and 1 nonpathogenic variant (c.3889G>A), were detected in the *ATP7B*. The variants consisted of 16 (64%) missense variants, 5 (20%) frameshift variants, 3 (12%) splice-site variants, and 1 (4%) large deletion mutation (Figure 2).

**Table 2:** Details of possible pathogenic variants of *ATP7B* gene identified in the 34 probands.

**Nucleotide change**	**Amino acid change**	**Exon**	**Area of protein**	**Mutation type**	**PolyPhen2^a^**	**Minor allele frequency (%) (EXAC)**	**Classification according to ACMG**	**Clinical significance**
c.1168A>G	p.I390V	2	MBD4	Missense	Benign	0.0018	4^*^BP, 5^*^BP	LB
c.1707+5G>A	n.a	4	MBD5/MBD6	Splicing	n.a	—	n.a	LP
c.1708-1G>C	n.a	5	MBD5/MBD6	Splicing	n.a	0.0028	1^*^PVS	P
c.2267C>G	p.A756G	8	TM3/TM4	Missense	Probably damaging	—	1^*^PM, 2^*^PM, 3^*^PP	LP
c.2333G>T	p.R778L	8	TM4	Missense	Probably damaging	0.0132	3^*^PS, 2^*^PM, 3^*^PP, 5^*^PP	P
c.2383C>T	p.L795F	9	TM4/A-domain	Missense	Probably damaging	0.0028	1^*^PM, 2^*^PM, 3^*^PP, 5^*^PP	LP
**c.2494A>C**	**p.K832Q**	**10**	**A-domain**	**Missense**	**Probably damaging**	**—**	**1** ^*^ **PM, 2** ^*^ **PM, 3** ^*^ **PM, 3** ^*^ **PP**	**LP**
c.2590_2593dup	p.T865SfsX3	11	A-domain	Frameshift	n.a	—	1^*^PVS	P
c.2621C>T	p.A874V	11	A-domain/TM5	Missense	Probably damaging	0.0068	3^*^PS, 1^*^PM, 2^*^PM, 3^*^PP	P
c.2662A>C	p.T888P	11	A-domain/TM5	Missense	Probably damaging	—	1^*^PM, 2^*^PM, 3^*^PP, 5^*^PP	LP
c.2755C>G	p.R919G	12	TM5	Missense	Possibly damaging	0.0039	3^*^PS, 2^*^PM, 3^*^PP, 5^*^PP	P
**c.2790_2792del**	**p.I930del**	**12**	**TM5**	**Frameshift**	**n.a**	**0.0032**	**1** ^*^ **PVS, 2** ^*^ **PM, 4** ^*^ **PM**	**LP**
c.2975C>T	p.P992L	13	TM6	Missense	Probably damaging	0.0036	3^*^PS, 2^*^PM, 3^*^PP, 5^*^PP	P
c.3316G>A	p.V1106I	15	N-domain	Missense	Probably damaging	0.0128	1^*^PM, 2^*^PM, 3^*^PP	LP
c.3451C>T	p.R1151C	16	N-domain	Missense	Probably damaging	0.0056	1^*^PM, 2^*^PM, 3^*^PP, 5^*^PP	LP
c.3517G>A	p.E1173K	16	N-domain	Missense	Probably damaging	0.0012	1^*^PM, 2^*^PM, 3^*^PP, 5^*^PP	LP
**c.3593T>C**	**p.V1198A**	**17**	**P-domain**	**Missense**	**Probably damaging**	**—**	**1** ^*^ **PM, 2** ^*^ **PM, 3** ^*^ **PP, 5** ^*^ **PP**	**LP**
c.3646G>A	p.V1216M	17	P-domain	Missense	Probably damaging	0.0085	1^*^PM, 2^*^PM, 3^*^PP, 5^*^PP	LP
**c.3659-3660insTGA**	**p.T1220-G1221insE**	**17**	**P-domain**	**Frameshift**	**n.a**	**—**	**1** ^*^ **PVS,2** ^*^ **PM, 4** ^*^ **PM**	**P**
c.3700del	p.V1234LfsX96	18	P-domain	Frameshift	n.a	—	1^*^PVS	P
**c.3724G>A**	**p.E1242K**	**18**	**P-domain**	**Missense**	**Probably damaging**	**—**	**1** ^*^ **PM, 2** ^*^ **PM, 3** ^*^ **PM, 3** ^*^ **PP**	**LP**
c.3884C>T	p.A1295V	18	P-domain	Missense	Probably damaging	—	1^*^PM, 2^*^PM, 3^*^PP, 5^*^PP	LP
c.3903+2T>G	n.a	18	P-domain	Splicing	n.a	—	1^*^PVS	P
c.4176dup	p.K1393EfsX15	21	after TM8	Frameshift	n.a	—	1^*^PVS	P

del, deletion; ins, insertion; n.a, not applicable; MBD, metal binding domain; TM, transmembrane domain; A-domain, actuator domain; P-domain, phosphorylation domain; N-domain, nucleotide-binding domain; EXAC, The Exome Aggregation Consortium; P, pathogenic; LP, likely pathogenic; VUS, uncertain significance; B, benign; LB, likely benign.

Novel mutations are shown in boldface letters.

a http://genetics.bwh.harvard.edu/pph2/index.shtml.

In addition to the mutations in the table, there was one large deletion mutation in this study.

**Figure 3:**
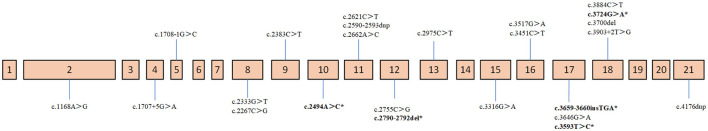
Distribution of mutations on *ATP7B* gene. Novel mutations are noted with a * behind.

Most of *the ATP7B* mutations were located in exon 18 (16.7%), exon 11 (12.5%), and exon 17 (12.5%; location of variants shown in Figure 3). The c.2333G>T (R778L, 39.06%) substitution at exon 8 was the most common mutation, followed by the c.2621C>T (A874V, 10.94%) at exon 11. There were 27 different variant combinations identified, among which the most common genotype was R778L/R778L, accounting for 14.71% (five out of 34) of these patients. Other common combinations of variants comprised R778L/A874V, R778L/P992L, and E1173K/ V1106I. Overall, among the 34 probands involved in this study, 29 patients (85.29%) were characterized with two potential disease-causing variants, and four patients (11.76%) were characterized with one potential disease-causing variant. Additionally, one patient carried three disease-causing variants. The genotype (c.1168A>G+c.1708-1G>C+c.2333G>T) consists of two “pathogenic” variants and one “likely benign” variant. The majority of the patients carrying two mutations were compound heterozygotes (23 out of 29), while others were homozygotes (six out of 29).

In our present study, the genetic analysis of the *ATP7B* gene identified five novel diseases (boldface letters in Table 2) causing mutations in these unrelated patients: c.3659-3660insTGA, c.3724G>A, c.2494A>C, c.3593T>C, and c.2790-2792delCAT. Out of the novel variants, three were missense variants, and the remaining two (one deletion mutation and one insertion variant) were frameshift variants. The novel mutation types were diverse, but they were all found in the compound heterozygous state, which was consistent with the autosomal recessive mode of Mendelian inheritance. The parents of three patients carrying the novel missense mutation (c.2494A>C, c.2790-2792delCAT, and c.3724G>A) also underwent relevant genetic testing. Based on family pedigrees analysis of three patients, these novel mutations were, respectively, present in the unaffected parent.

According to the ACMG guidelines, two frameshift variants (one deletion mutation and one insertion variant) could be evaluated as “pathogenic” (PVS1 + PM2 + PM4). Both the two missense variants (c.3593T>C and c.3724G>A) are located in the hotspot mutation region of *ATP7B*. *In silico* pathogenicity prediction programs (Mutation Taster, PolyPhen-2) indicated all novel missense mutations have damaging effects on the function of proteins. Therefore, c.3593T>C and c.3724G>A could be classified as “likely pathogenic variants.” The novel heterozygous mutation c.2494A>C was detected in exon 10 of the *ATP7B* gene in patient 14. Genetic analysis has shown that the heterozygous mutation c.2494A>C was inherited from his father and that the heterozygous mutation c.2333G>T was inherited from his mother. According to the family history and the ACMG guidelines, c.2494A>C could also be evaluated as “likely pathogenic variants” (PM1 + PM2 + PM3 + PP3).

## 4. Discussion

In this study, *ATP7B* analysis in 34 cases enrolled through various families revealed a total of 25 underlying disease-causing mutations. Data retrieved through studies have shown that the mutational spectrum of the *ATP7B* gene varies among different populations, i.e., R778L is mostly prevalent in Korean and Chinese patients, H1069Q is prevalent among European descent (Europe and North America), R778W is predominantly reported from the Indian population (13). Despite the small number of WD patients we examined, the most common mutation had been confirmed to be c.2333G>T, which was in accordance with previous studies. In a recently reported Chinese cohort, the mutations p.R778L (28.96%) followed by p.P992L (13.82%) and p.A874V (5.99%) were the most frequent variants (7). However, in our present cohort, the frequencies of p.R778L (39.06%), p.A874V (10.94%), p.V1106I (7.81%), and p.P992L(6.25%) were different from those previously reported. In fact, this difference may be explained by the small sample size and the geographic area of patients (most of our cases were from Henan Province).

Relevant data associated with *ATP7B* mutations indicate that the majority are missense mutations (1, 14). In our research, the 25 variants consisted of 16 missense variants, accounting for 64%, in agreement with those found in previous reports. In addition, we detected five frameshift variants and three splicing variants. Notably, we also identified one large deletion mutation (located in exon 2) using multiplex ligation-dependent probe amplification (MLPA). Although most species of *ATP7B* variants could be identified using the next-generation sequencing (NGS) technology (15), a further technique is required to be a supplement for a few of the patients whose genetic diagnosis could not be successfully established. As reported by Chen et al., for index cases, NGS only identified a heterozygous variant in *ATP7B* analysis, with MLPA allowed to discover other rare variants including large *ATP7B* deletion/duplication (16, 17). In our current study, patient 1 was first identified with a single heterozygous missense mutation detected by NGS during a workup for liver damage. However, additional clinical features including reduced serum ceruloplasmin and neurological performance (such as dystonia) prompted further analysis of *ATP7B*. By using MLPA, a gross deletion in exon 2 was found in the patient whose other mutation was a missense mutation (p.A874V) in exon 11. Therefore, MLPA could be applied beyond NGS in early and timely diagnosis for affected individuals.

Pathologically, we described the thinning of the glomerular basement membrane in WD patients for the first time. Penicillamine was mentioned to cause membranous nephropathy in previous reports (10). The pathological features of membranous nephropathy mainly include the thickening of the glomerular capillary basement membrane, granular deposits in the glomerular capillary wall, and fusion of podocytes (18). However, the renal electron microscopy results showed diffuse thinning of the basement membrane instead of thickening in our current study. It is difficult to explain such results in terms of membranous nephropathy. Therefore, we considered that the thinning of the glomerular basement membrane in patient 2 was not related to penicillamine. Recent research had demonstrated that diffuse thinning of the basement membrane was commonly seen in hereditary kidney disease. Thin basement membrane nephropathy (TBMN) resulted from pathogenic variants in *COL4A3/COL4A4/COL4A5* (19). Nevertheless, no mutations in TBMN-related genes were detected in the genetic analysis of this patient. Therefore, we speculated that diffuse thinning of the basement membrane may be a rare pathologically damaging feature of Wilson's disease. It has been shown that excessive copper deposition in many organs such as the liver and central nervous system might lead to the impairment of the physiological functions of the affected organs (20, 21). Similarly, this renal pathological feature may be explained by excessive copper deposition on the glomerular basement membrane. The limitations of our current study included the small sample size and the lack of data on the level of copper in the kidney. Four mutations in two patients with renal involvement were located in the phosphorylation domain (c.3659-3660insTGA in patient 2 and c.3724G>A in patient 10), transmembrane domain (c.2333G>T in patient 2), and actuator domain (c.2621C>T in patient 10), respectively. We could not draw out the hotspot mutation location for kidney impairment for Wilson's disease because of the limitation of the case number.

Treatment of WD was recommended to be initiated as early as possible, and individualized and lifelong therapy was emphasized (22). There are some major pathways for the general management of medical treatment, supporting therapy, liver transplantation, etc. (6, 23). Medical treatment of copper-chelators (such as D-penicillamine, sodium dimercaptosulphonate, dimercaptosuccinic acid, and trientine) or zinc could greatly improve the outcomes of patients with Wilson's disease. However, liver transplantation was recommended for those presenting with decompensated cirrhosis and fulminant hepatic failure (24). It had been reported that liver transplantation could improve copper metabolism and reduce the manifestations of impaired liver function. In addition, some studies claimed that liver transplantation relieved neurological symptoms (25). In our present cohort, of those patients with liver involvement, two pediatric patients underwent liver transplantation for decompensated cirrhosis. The levels of biochemical indicators (such as serum ceruloplasmin and albumin) and the clinical manifestations (such as weakness, poor appetite, and edema of both lower extremities) were significantly improved after surgery in line with those found in other studies. During our average 20-month postoperative follow-up, the two cases got considerable improvement in their condition. However, long-term survival after liver transplantation would require longer follow-ups and larger study cohorts. In addition to the above treatment modalities, it is essential for all patients to adhere to a low-copper diet to control their condition (26).

## 5. Conclusion

In conclusion, our study identified five novel mutations that broadened the spectrum of pathogenic *ATP7B* variants. Among these novel variants, three missense variants were classified as “likely pathogenic variants,” and two frameshift variants were classified as “pathogenic variants” in this stage. We observed one large deletion mutation by MLPA, which suggested that MLPA could be applied beyond NGS in molecular diagnosis for affected individuals. Moreover, diffuse thinning of the basement membrane may be a rare pathologically damaging feature of Wilson's disease. Furthermore, liver transplantation had been observed to be a promising treatment modality in this study. However, the small sample size, lack of follow-up, and its retrospective nature indicate that further study is necessary.

## Data availability statement

The original contributions presented in the study are publicly available. This data can be found here: European Molecular Biology Laboratory's European Bioinformatics Institute (EMBL-EBI), European Variation Archive (EVA), https://www.ebi.ac.uk/eva/, PRJEB65581.

## Ethics statement

Written informed consent was obtained from the individual(s), and minor(s)' legal guardian/next of kin, for the publication of any potentially identifiable images or data included in this article.

## Author contributions

HX designed the study and drafted the manuscript. HL contributed to the study design and drafted the manuscript. XC contributed to the study design and revised the manuscript. YL and GX coordinated the implementation of research activities. YW and RH carried out data curation and analysis. All authors have read and agreed to the published version of the manuscript.
