# Erratum: Novel mutations in ATP7B in Chinese patients with Wilson's disease and identification of kidney disorder of thinning of the glomerular basement membrane

**DOI:** 10.3389/fneur.2023.1290996

**Published:** 2023-09-20

**Authors:** Frontiers Production Office

**Affiliations:** Frontiers Media SA, Lausanne, Switzerland

**Keywords:** Wilson's disease, *ATP7B* gene, novel mutations, renal pathological, liver transplantation

Due to a production error, a correction has been made to the Data Availability Statement. The paragraph now reads:

“The original contributions presented in the study are publicly available. This data can be found here: European Molecular Biology Laboratory's European Bioinformatics Institute (EMBL-EBI), European Variation Archive (EVA), https://www.ebi.ac.uk/eva/, PRJEB65581.”

The publisher apologizes for this mistake. The original article has been updated.

